# Supplementary material for: Immune checkpoint blockade in triple negative breast cancer influenced by B cells through myeloid-derived suppressor cells
Source: Commun Biol. 2021 Jul 12;4:859. doi: 10.1038/s42003-021-02375-9 (PMC8275624; doi:10.1038/s42003-021-02375-9)
Supplement: Supplementary file 2 — Supplementary Information [file 42003_2021_2375_MOESM2_ESM.pdf]

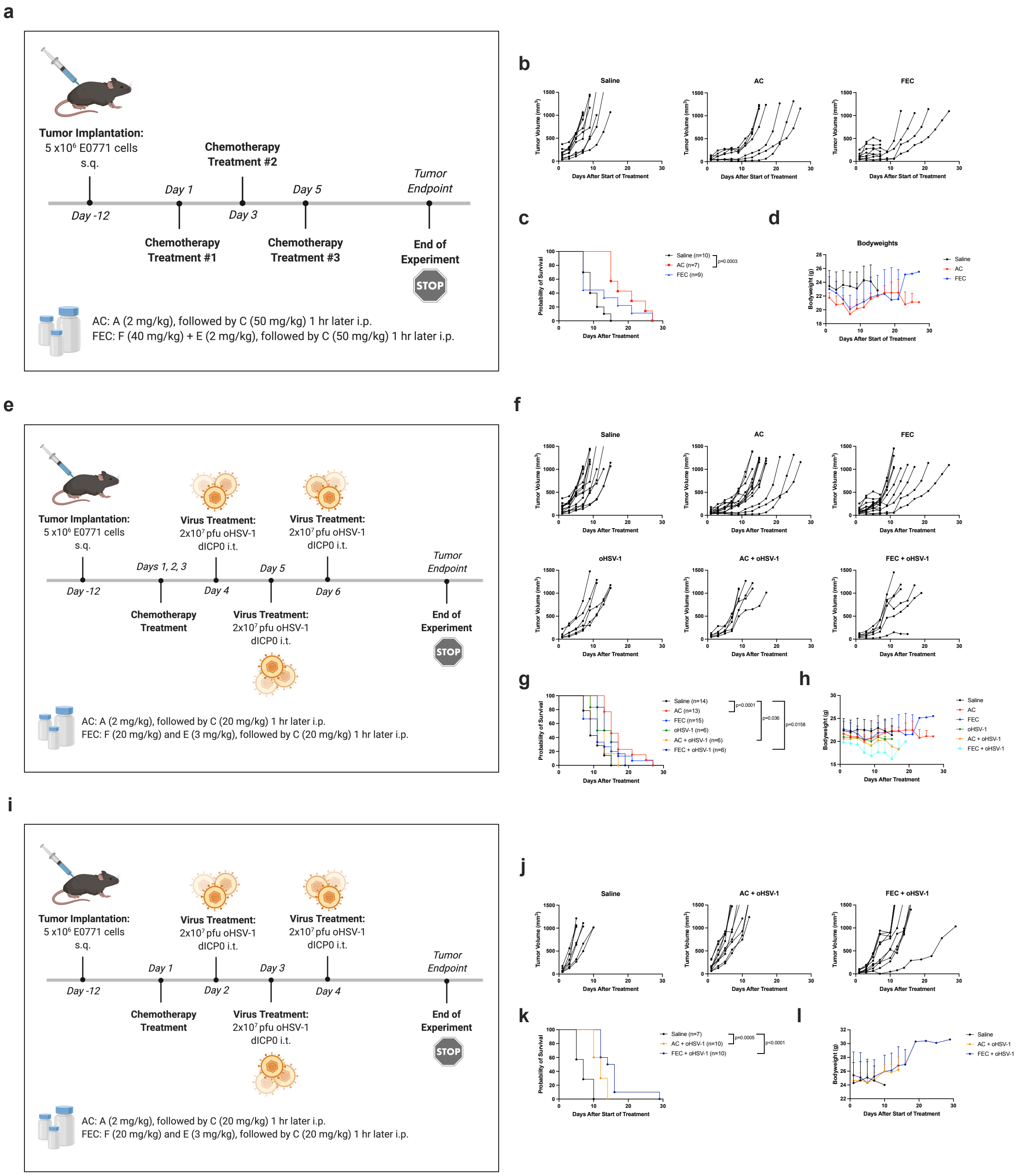

Supplementary Figure 1. Preliminary dose optimization studies for AC and FEC chemotherapy regimens

(**a**)(**e**)(**i**) C57/Bl6 mice bearing E0771 tumors were treated with saline, AC, FEC, oHSV-1, AC + oHSV-1 or FEC + oHSV-1. Each treatment schedule outlines a separate, successive experiment. *Created using BioRender.com. (**b**)(**f**)(**j**) Tumor volumes were measured every 2-3 days from the start of treatment until mice reached endpoint. Each line represents an individual mouse within the group. (**c**)(**g**)(**k**) Kaplan-Meier survival curves of each group. (**d**)(**h**)(**l**) Average bodyweights for all groups. *Mantel-Cox test was used for statistical analyses. Error bars are representative of the standard deviation. Note: Survival statistics are not an accurate measure of therapeutic efficacy for experiments 1 and 2 (c and g), as many mice were sacrificed due to extreme weight loss, rather than a lack of therapeutic efficacy.

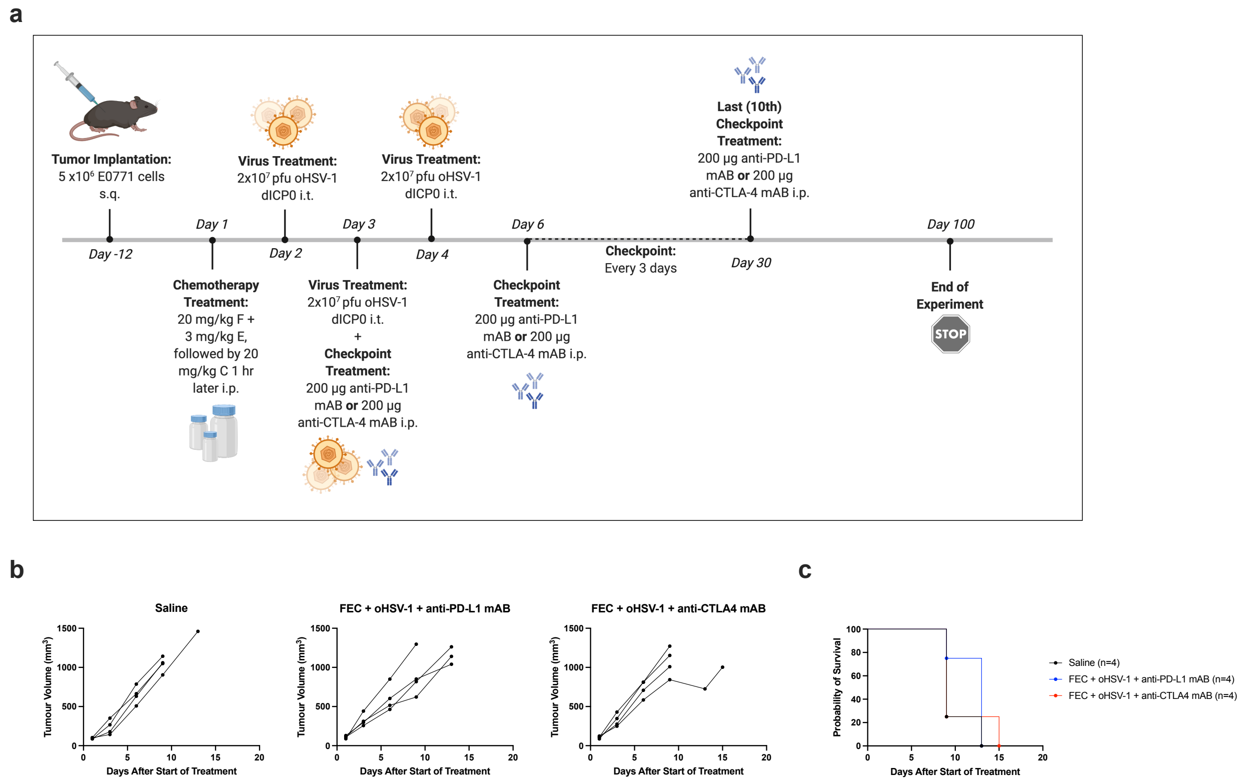

Supplementary Figure 2. Administration of singular checkpoint antibodies shows no therapeutic efficacy

(**a**) C57/Bl6 mice bearing E0771 tumors were treated with saline or FEC + oHSV-1 + CP (anti-CTLA4 or anti-PD-L1). *Created using BioRender.com. (**b**) Tumor volumes were measured every 2-3 days from the start of treatment until mice reached endpoint (tumor volume = 1000 mm^3). Each line represents an individual mouse within the group. (**c**) Kaplan-Meier survival curves of each group. *Mantel-Cox test was used for statistical analyses.

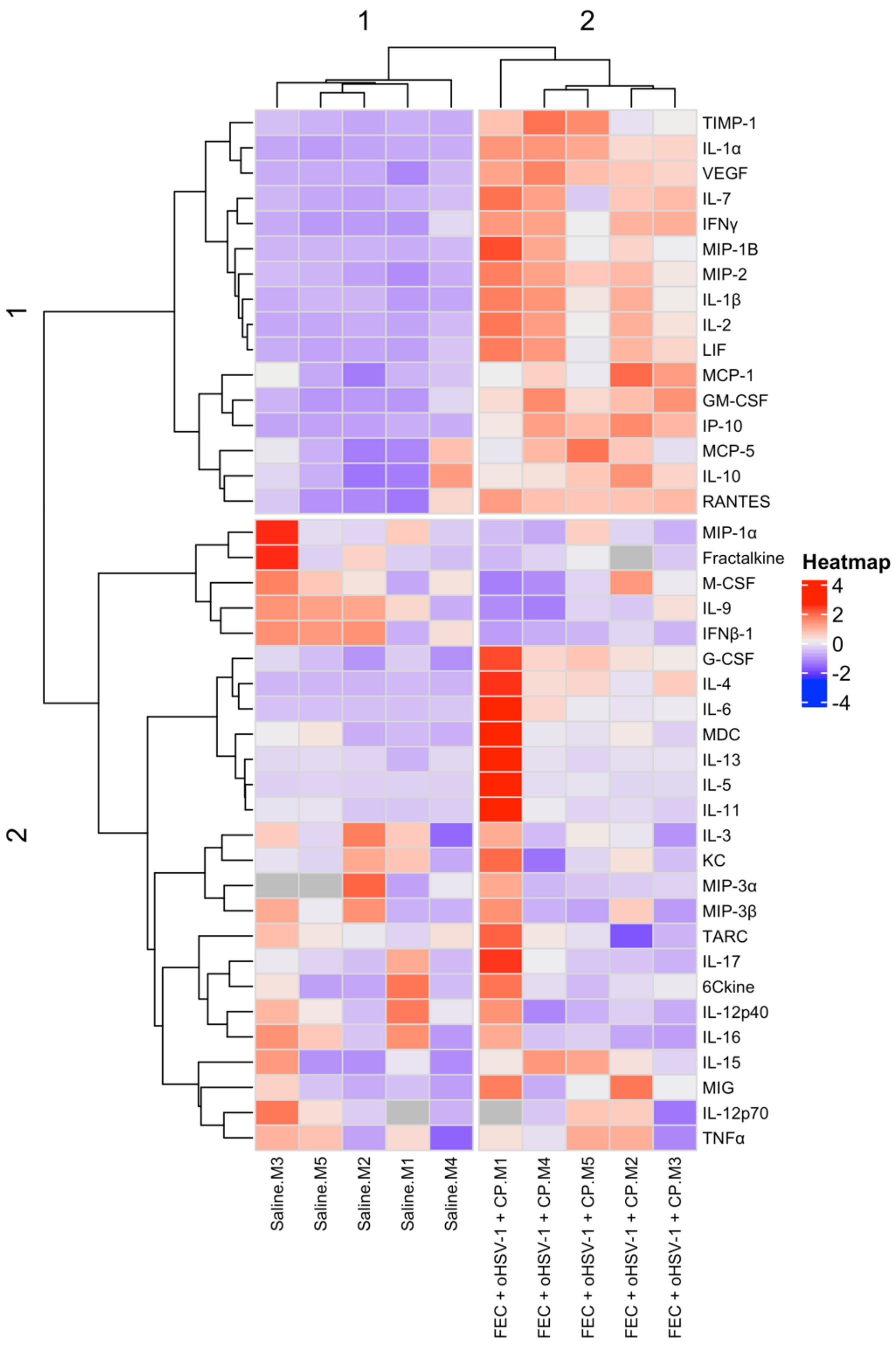

Supplementary Figure 3. FEC + oHSV + CP significantly changes the expression of many cytokines in tumor-bearing mice

E0771 tumors were grown in C57/Bl6 mice. Mice were sacrificed on day 10 and tumors flash frozen. Tumor lysates were made and sent for cytokines expression analysis. Heat map showing the differential expression levels (concentration, pg/mL) of a panel of cytokines. All values are reported as a concentration (pg/mL).

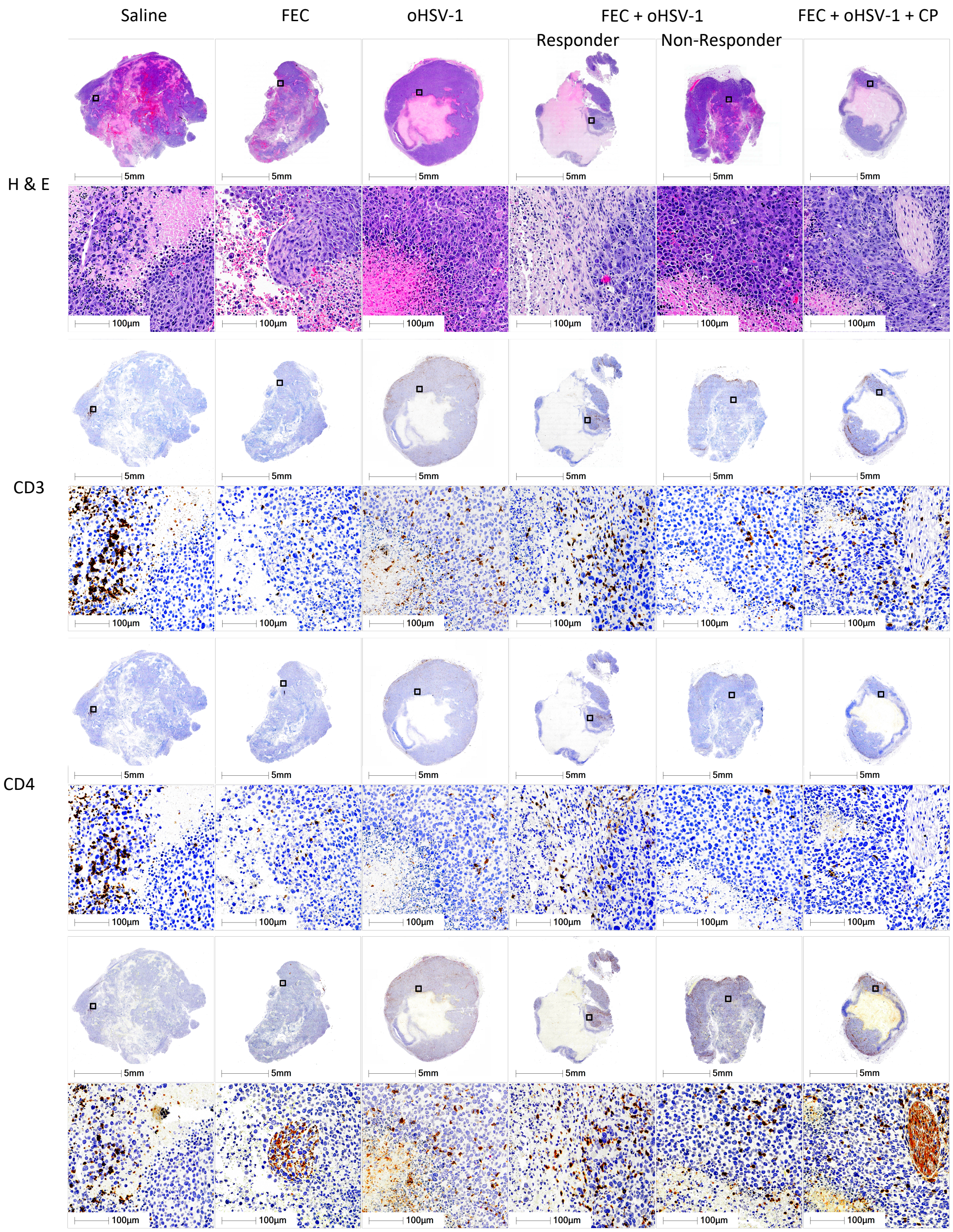

Supplementary Figure 4. Immunohistochemistry analysis on day 10 shows that FEC + oHSV-1 and FEC + oHSV-1 + CP treatments induce TIL infiltration

C57/Bl6 mice bearing E0771 tumors were treated with saline, FEC, oHSV-1, FEC + oHSV-1 or FEC + oHSV-1 + CP and tumors were harvested on day 10 (n=5 per group). Tumors were sectioned and stained with H&E for pathological analysis. Sections were then further stained with antibodies for CD3, CD4, CD8α and FOXP3. Representative images for tumors are shown. Each image shows a whole section of an individual tumor.

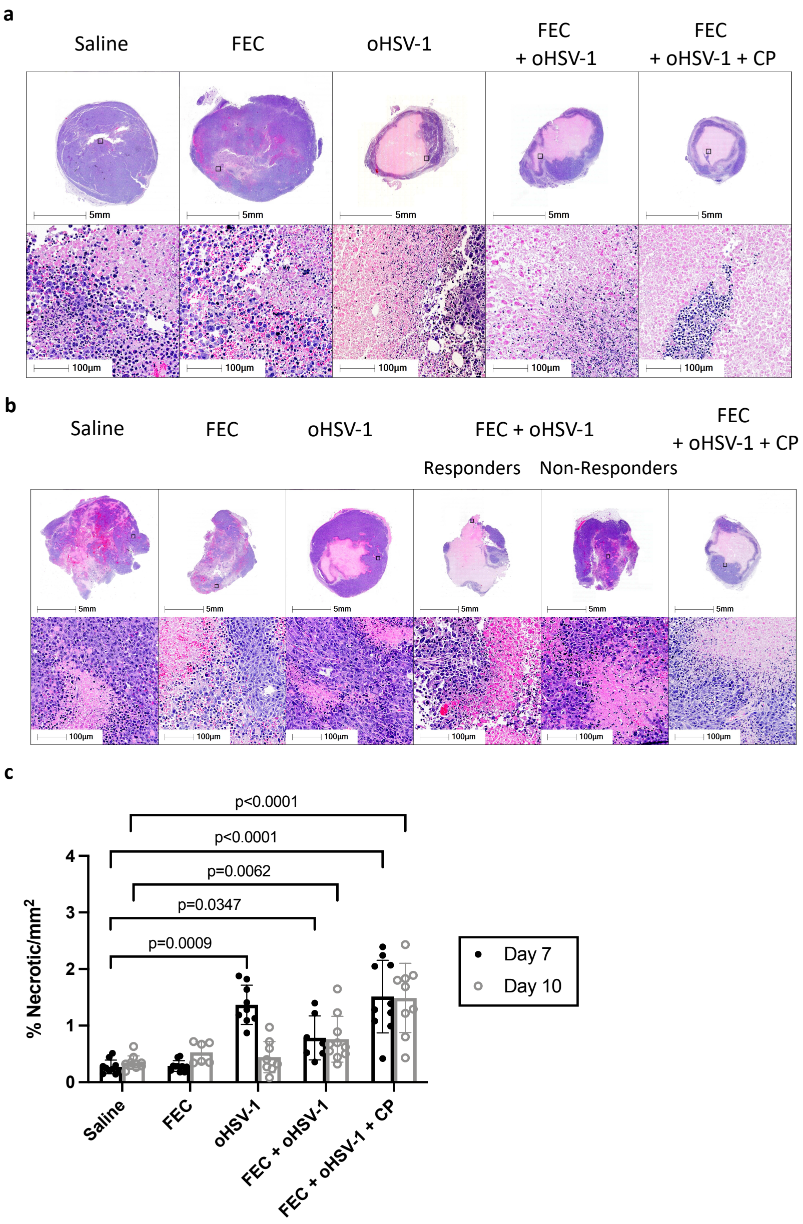

Supplementary Figure 5. H&E staining reveals increased necrotic tissue in FEC + oHSV-1 + CP treated mice

C57/Bl6 mice bearing E0771 tumors were treated with saline, FEC, oHSV-1, FEC + oHSV-1 or FEC + oHSV-1 + CP and tumors were harvested on days 7 and 10. Tumors were sectioned and stained with H&E for pathological analysis. (**a**) Representative images for tumors harvested on day 7. Each image shows a whole section of an individual tumor. (**b**) Representative images for tumors harvested on day 10. Each image shows a whole section of an individual tumor. (**c**) Whole tumor sections were scanned, and viable and necrotic cells were quantified using HALO quantification software. Each symbol represents an individual mouse within that group. Two-tailed paired t test was used for statistical analyses. Error bars are representative of the standard deviation.

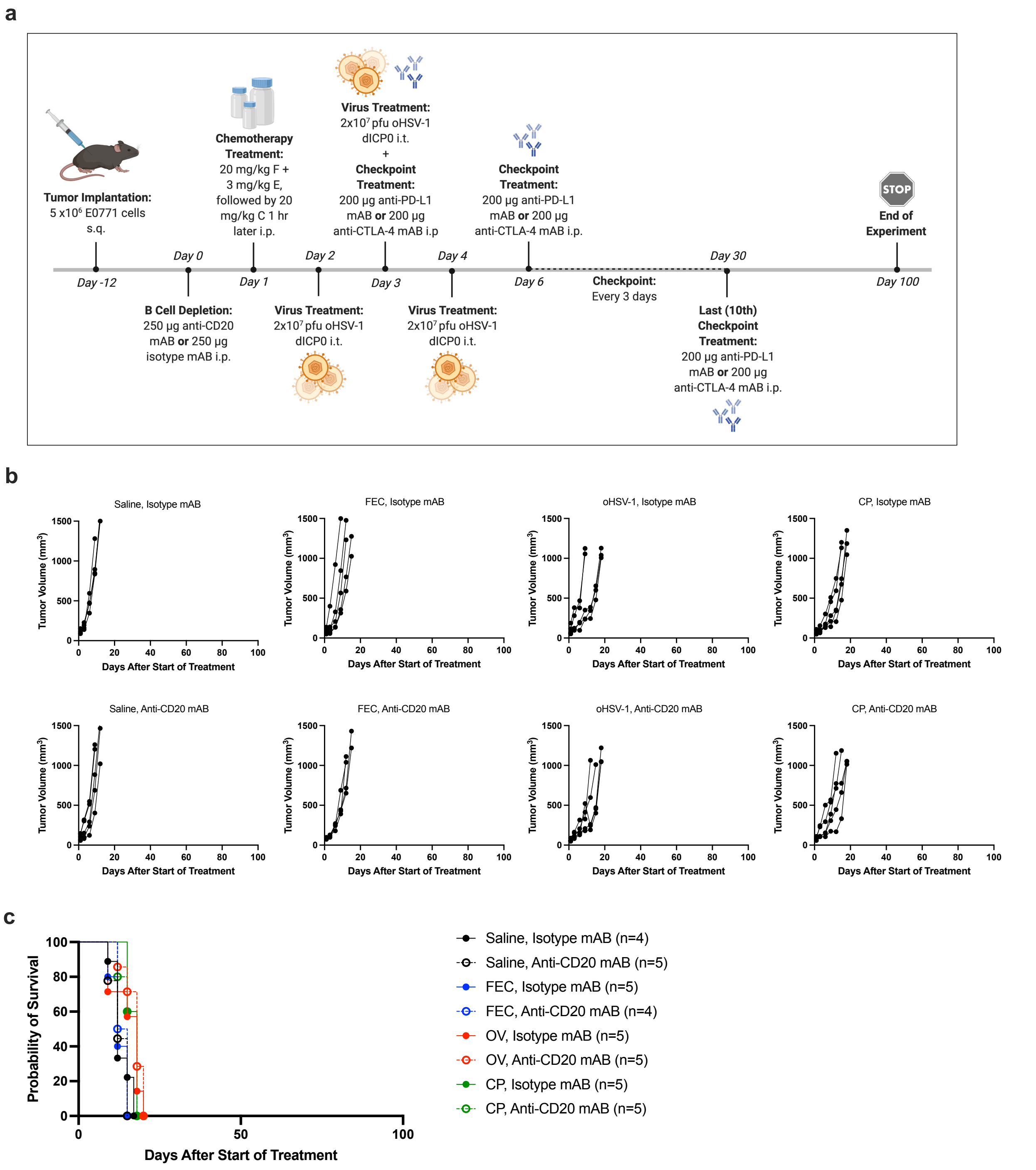

Supplementary Figure 6. B cells are required for therapeutic efficacy of combination therapies

(**a**) C57/Bl6 mice bearing E0771 tumors were treated with anti-CD20 mAB or isotype mAB 24hr prior to the start of treatment. Groups of mice were then treated with saline, FEC, oHSV-1, CP, FEC + oHSV-1 or FEC + oHSV-1 + CP. *Created using BioRender.com. (**b**) Tumor volumes were measured every 2-3 days from the start of treatment until mice reached endpoint (tumor volume > 1000 mm^3). Each line represents individual mice within the group. (**c**) Kaplan- Meier survival curves of each group.

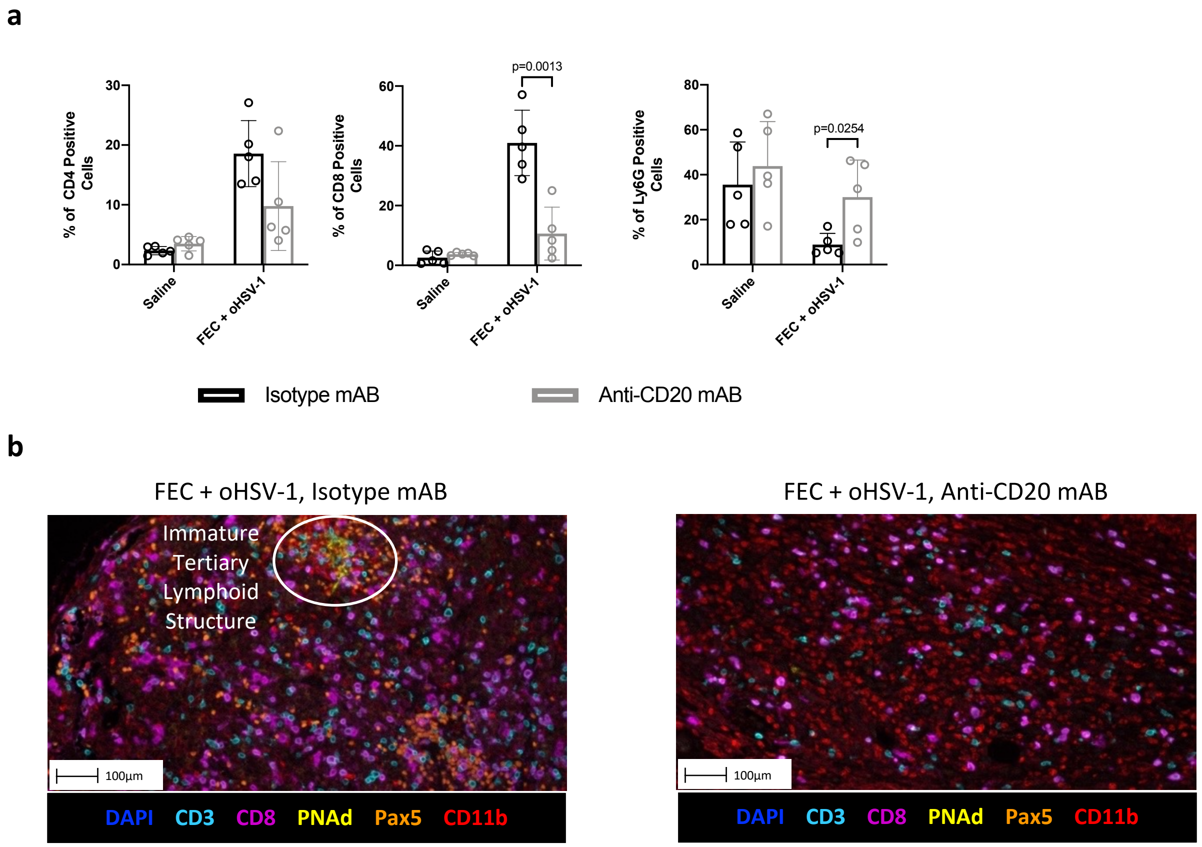

Supplementary Figure 7. Depletion of B cells results in disruption of immune cell organization

C57/Bl6 mice bearing E0771 tumors were treated with anti-CD20 mAB or isotype mAB 24hr prior to the start of treatment. Groups of mice were then treated with saline or FEC +oHSV-1. Mice were sacrificed on day 10 and tumors were harvested for analysis. (**a**) Whole tumor sections were scanned and quantified using HALO quantification software. Each symbol represents an individual mouse within that group. (**b**) Multi-panel IF staining with DAPI, as well as antibodies to stain for CD3 (light blue), CD8 (magenta), PNAd (yellow), Pax5 (orange) and CD11b (red). *Two-tailed unpaired t test was used for statistical analyses. Error bars represent the standard deviation.

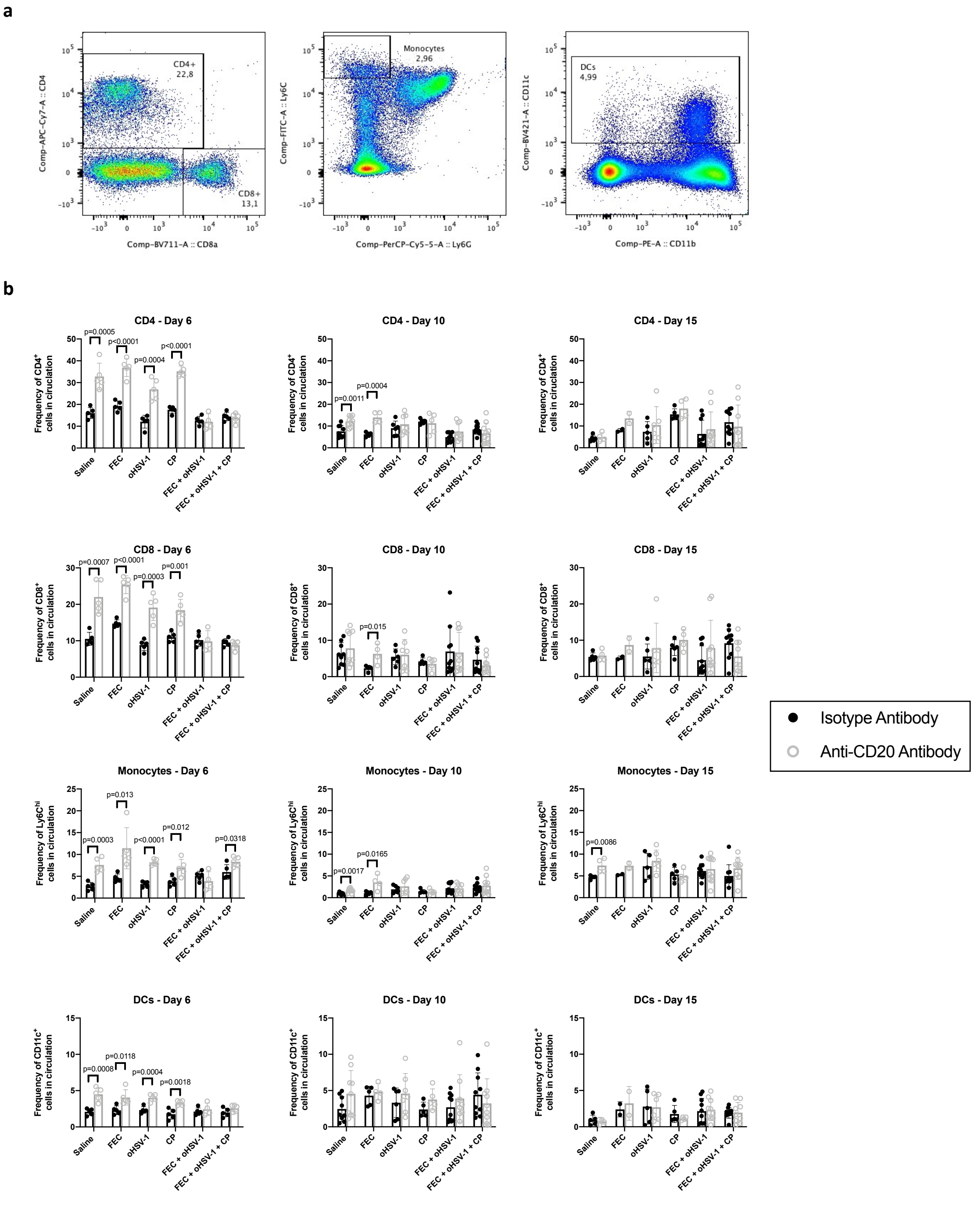

Supplementary Figure 8. Immune analysis of CD4$^+$, CD8$^+$, monocytes and DCs

C57/Bl6 mice bearing E0771 tumors were treated with saline, FEC, oHSV-1, CP, FEC + oHSV-1 or FEC + oHSV-1 + CP. Half of the mice were treated with an isotype mAB and half were treated with an anti-CD20 mAB. Blood was drawn on days 6, 10 and 15 and analyzed via flow cytometry. (**a**) Representative flow plot showing the gating strategy for CD4$^+$, CD8$^+$, monocytes (Ly6C^{hi}Ly6G$^-$ cells) and DCs (CD11c$^+$). (**b**) Bar plots showing the frequency of immune cells in circulation across all timepoints. Two-tailed unpaired t test was used for statistical analyses. Error bars are representative of standard deviation.

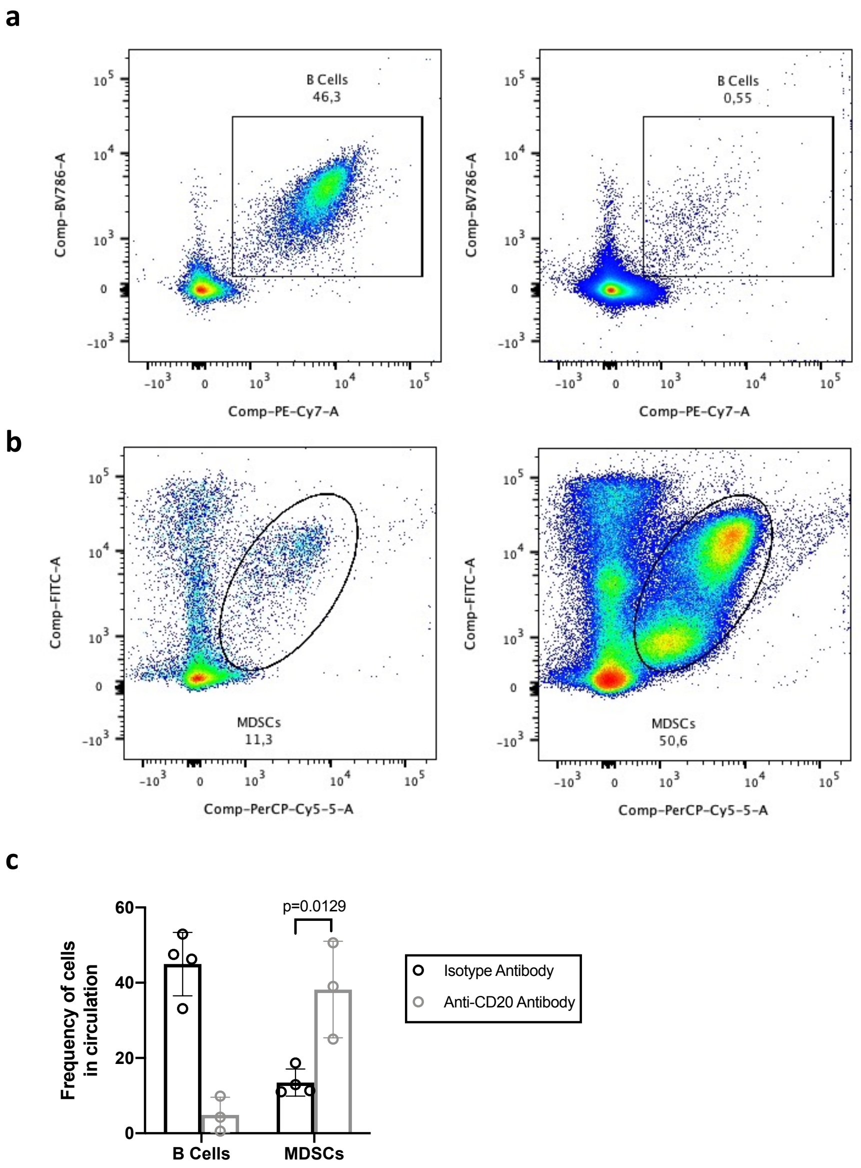

Supplementary Figure 9. Immune analysis of B cells and MDSCs in PY230 tumors

C57/Bl6 mice bearing PY230 tumors were treated with FEC + oHSV-1 + CP. Half of the mice were treated with an isotype mAB and half were treated with an anti-CD20 mAB. Blood was drawn on day 29 and analyzed via flow cytometry. (**a**) Representative flow plots showing the gating strategy for B cells ($CD19^{+}B220^{+}$ cells). The left plot shows a mouse treated with isotype mAB and the right plot shows a mouse treated with anti-CD20 mAB. (**b**) Representative flow plots showing the gating strategy for MDSCs ($Ly6C^{int}Ly6G^{hi}$ cells). The left plot shows a mouse treated with isotype mAB and the right plot shows a mouse treated with anti-CD20 mAB. (**c**) Frequency of B cells and MDSCs, as determined by flow cytometry. Two-tailed unpaired t test was used for statistical analyses. Error bars are representative of standard deviation.

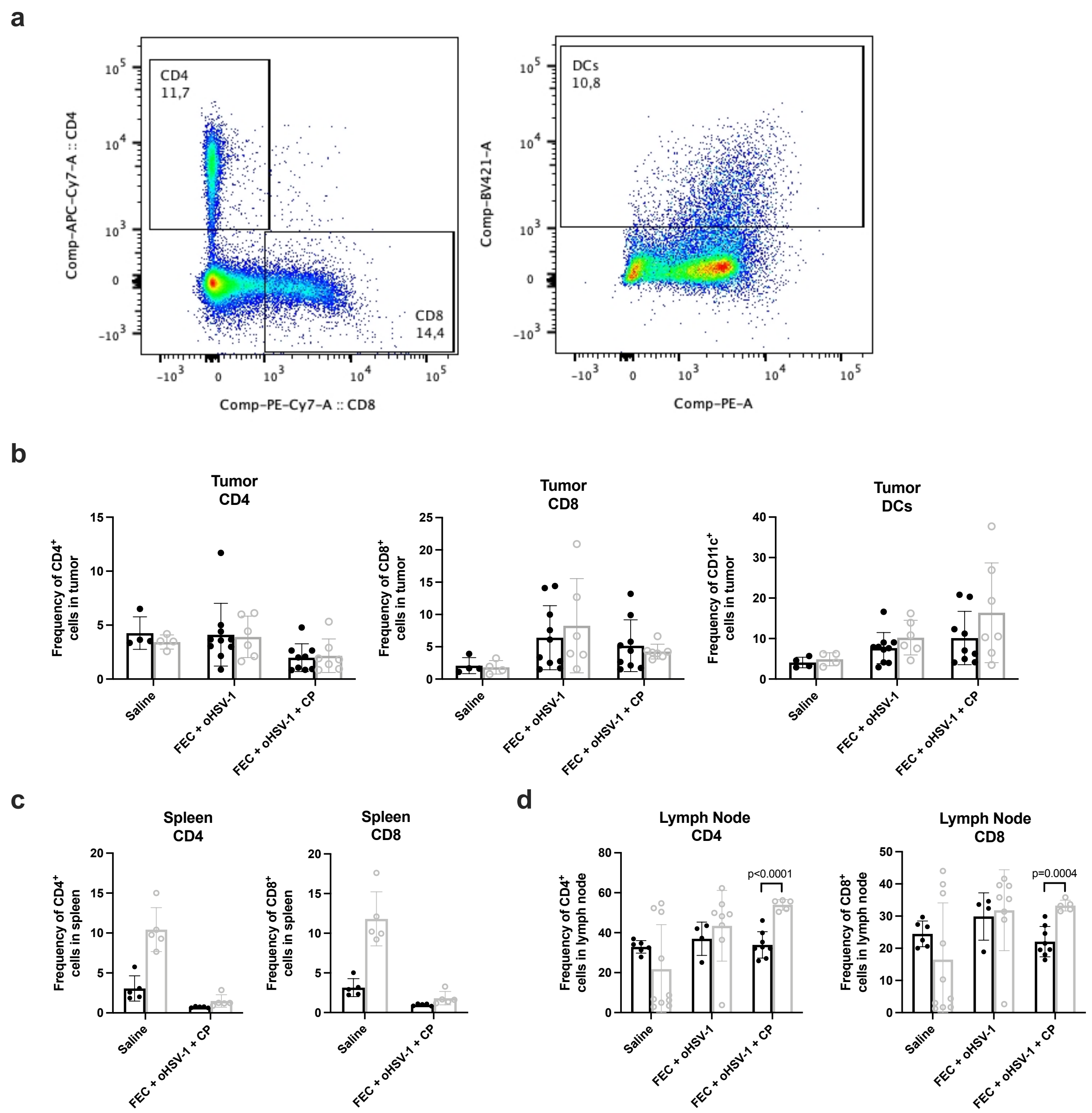

Supplementary Figure 10. Immune analysis of CD4$^+$, CD8$^+$ and DCs in the tumor, spleen and TDLN

C57/Bl6 mice bearing E0771 tumors were treated with saline, FEC + oHSV-1 or FEC + oHSV-1 + CP. Half of the mice were treated with an isotype mAB and half were treated with an anti-CD20 mAB. Mice were sacrificed and organs harvested on day 10 for analysis via flow cytometry. (**a**) Representative flow plot showing the gating strategy for CD4$^+$, CD8$^+$ and DCs (CD11c$^+$). (**b**) Bar plots showing the frequency of immune cells in the tumor. (**c**) Bar plots showing the frequency of immune cells in the spleen. (**d**) Bar plots showing the frequency of immune cells in the TDLN. *Two-tailed unpaired t test was used for statistical analyses. Error bars are representative of standard deviation.
