# Supplementary material for: Immune checkpoint blockade in triple negative breast cancer influenced by B cells through myeloid-derived suppressor cells
Source: Commun Biol. 2021 Jul 12;4:859. doi: 10.1038/s42003-021-02375-9 (PMC8275624; doi:10.1038/s42003-021-02375-9)
Supplement: Supplementary file 3 — Description of Supplementary Files [file 42003_2021_2375_MOESM3_ESM.pdf]

Description of Additional Supplementary Files

File Name: Supplementary Data 1
Description: Differentially expressed genes associated with B cell pathways**.** Values are representative of fold change. F = FEC, O = oHSV-1, S = Saline, BCR = B cell receptor, KEGG = Kyoto Encyclopedia of Genes and Genomes, Sino = Sino Biological.

File Name: Supplementary Data 2
Description: Antibody information

File Name: Supplementary Data 3
Description: Raw data for all figures.
